# Predicting secondary surgery after operative fixation of olecranon fractures: a model using data from 800 patients

**DOI:** 10.1016/j.jseint.2021.04.014

**Published:** 2021-05-21

**Authors:** Kaare S. Midtgaard, Frede Frihagen, Grant J. Dornan, Marius Coucheron, Carina Fossåen, Dag Grundel, Christopher Gundersen, Stian Kristoffersen, Erik Sundqvist, Leonore Wünsche, Jan Erik Madsen, Gunnar B. Flugsrud

**Affiliations:** a Norwegian Armed Forces Joint Medical Services, Oslo, Norway; b Division of Orthopaedic Surgery, Oslo University Hospital, Oslo, Norway; c Institute of Clinical Medicine, University of Oslo, Oslo, Norway; d Department of Orthopaedic Surgery, Østfold Hospital Trust, Grålum, Norway; e Steadman Philippon Research Institute, Vail, CO, USA; f Department of Orthopedic Surgery, Diakonhjemmet Hospital, Oslo, Norway; g Department of Orthopedic Surgery, Stavanger University Hospital, Stavanger, Norway; h Department of Orthopedic Surgery, Nordlandssykehuset, Bodø, Norway; i Department of Orthopedic Surgery, Baerum Hospital, Vestre Viken Hospital Trust, Baerum, Norway; j Department of Orthopedic Surgery, Telemark Hospital, Skien, Norway; k Department of Orthopedic Surgery, St. Olavś Hospital, Trondheim, Norway

**Keywords:** Olecranon fracture, Elbow injury, Risk factors, Elbow fracture, Tension band wiring, Plate fixation

## Abstract

**Background:**

High rates of secondary surgery after fixation of olecranon fractures have been reported. Identification of risk factors can aid surgeons to reduce complications leading to additional surgical procedures.

**Methods:**

Olecranon fractures treated at seven hospitals from 2007 to 2017 were identified, and the radiographs were classified. Isolated, displaced olecranon fractures treated operatively with tension band wiring (TBW) or precontoured plate fixation (PF) were reviewed. Adjusted risk factors for secondary surgery were analyzed, and a multivariable predictive model for secondary surgery was built.

**Results:**

After the initial review of 1259 olecranon fractures, 800 isolated, displaced olecranon fractures met the inclusion and exclusion criteria. The distribution of two-part and multifragmented fractures was equal. TBW was used in 636 patients and PF in 164 patients. Multifragmentation was a significant variable influencing preference for PF. Secondary surgery was performed in 41% patients and symptomatic hardware removal was the most frequent primary indication. In both the TBW and PF group, the rates of major complications leading to secondary surgery were 13% (*P* = .96). The adjusted risk of secondary surgery was lower with increasing age (odds ratio by 10 years increments, 0.74; 95% confidence interval, 0.68-0.80, *P* < .01). Compared with PF, TBW with transcortical K-wires (odds ratio, 2.06; 95% confidence interval, 1.36-3.14; *P* < .01) and TBW with intramedullary K-wires (odds ratio, 4.32; 95% confidence interval, 2.16-8.86, *P* < .01) had significantly higher adjusted risk of secondary surgery.

**Conclusion:**

Surgeons preferred to use PF in younger patients and multifragmented fractures. Patients should be counseled that secondary surgery is common after surgical fixation of olecranon fractures. Symptomatic hardware removal was the most frequently reported reason for secondary surgery and more frequent after TBW. When using TBW, intramedullary K-wire positioning should be avoided. The rate of major complications leading to secondary surgery was similar in the TBW and PF groups. Overall, the risk of subsequent secondary surgery was higher in younger patients and patients treated with TBW.

Although olecranon fractures are one of the most commonly occurring elbow injuries, there is a dearth of literature. Only seven randomized trials, all with small sample sizes totaling 311 patients, have studied the outcomes after surgical treatment of isolated, displaced olecranon fractures.^4^^,^^12^ In summary, good outcomes can be expected after surgical treatment of olecranon fractures, but the reported rate of secondary surgery is between 25% and 88%.^2^^,^^4^^,^^5^^,^^12^

Currently, tension band wiring (TBW) is considered the standard treatment in isolated, displaced two-part olecranon fractures (Fig. 1). The implant is affordable,^6^^,^^19^ requires less time in the operating theater,^8^ and the implant has comparable biomechanical and clinical results with that of plate fixation (PF).^4^^,^^8^^,^^13^ However, there are technical pitfalls associated with TBW.^18^ The efficacy of TBW is dependent on adequate tensioning of the metal wire cerclage. Inadequate tension can result in secondary displacement and excessive tensioning can weaken the TBW construct.^13^ Hence, PF has emerged as a reliable alternative that may be easier to standardize. Another argument in favor of PF is that the rate of secondary surgery seems to be lower after PF of olecranon fractures.^4^^,^^8^ However, it has been demonstrated that TBW is more cost-effective compared with PF even when higher expected reoperation rate is taken into account.^6^^,^^19^ Moreover, serious complications such as deep infections seem to be more frequent after PF.^4^

**Figure 1:**
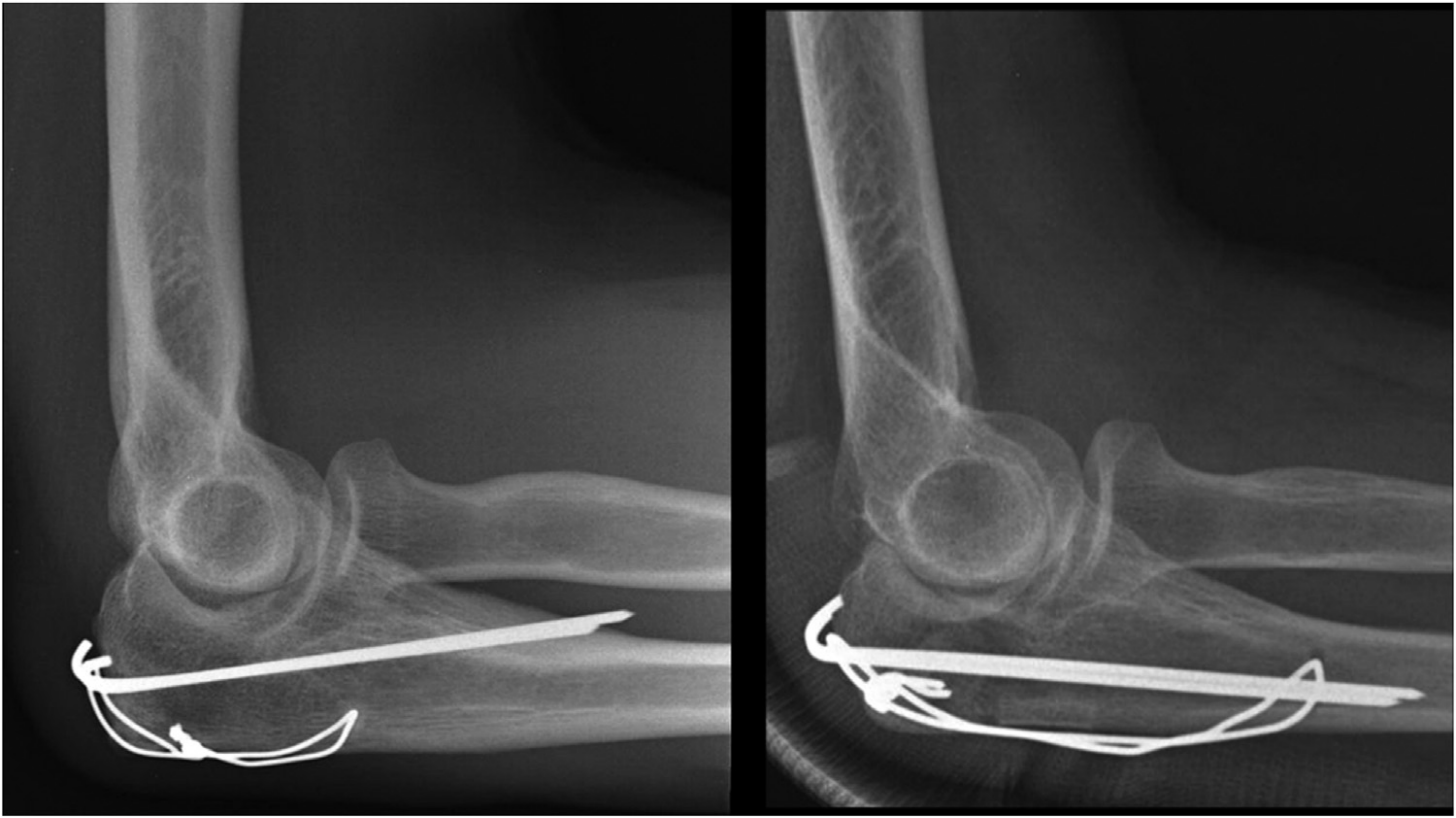
Tension band wiring with transcortical K-wires penetrating the anterior cortex (*Left*). Tension band wiring with intramedullary K-wires (*Right*).

(Reprinted with permission from Elsevier.^20^)

Numerous alternative fixation methods have been developed in an effort to reduce the rate of secondary surgery, including all-suture techniques,^1^ intramedullary rods,^16^ tension plates,^7^ and olecranon sleds.^11^ Nevertheless, TBW and PF remain the preferred methods for operative treatment of isolated, displaced olecranon fractures.^12^

Several factors influence the rate of secondary surgery. In the randomized controlled trial by Duckworth et al,^4^ 50% in the TBW group required symptomatic hardware removal compared with 22% in the PF group. In contrast, Claessen et al^2^ did not find that type or configuration of implant influenced secondary surgery. However, they did suggest that young age and female gender were associated with higher risk of secondary surgery.

The aims of this study were to describe how patient- and injury-related factors influence the choice of implant in fixation of isolated olecranon fractures. Furthermore, the aim was to describe risk factors for complications leading to secondary surgery and build a predictive model for subsequent surgery after fixation with TBW and PF.

## Methods

Ethical board approval was obtained from the Norwegian Regional Committees for Medical and Health Research Ethics (REK, 2018/2204). Data collection was performed at seven hospitals from all administrative health regions in Norway. Patients diagnosed with olecranon fracture (International Classification of Diseases 10th Revision [ICD-10] code S52.0) between January 2007 and December 2017 were identified in the digital patient registers. In addition, operating theater tracking tools were reviewed to identify misdiagnosed patients with olecranon fractures.

The inclusion criteria were isolated, displaced two-part (Mayo 2A)^14^ and multifragmented (Mayo 2B)^14^ olecranon fractures treated operatively with either TBW or precontoured PF. Patients younger than 16 years of age at the time of surgery, as well as patients with bilateral olecranon fractures or those treated nonoperatively, were excluded. Patients with concomitant ipsilateral coronoid process fractures, radial head fractures, or distal humerus fractures were excluded. PF other than precontoured olecranon plates was excluded. At the study centers, new radiographs were obtained routinely at 4-8 weeks and examined in the outpatient clinic. Patients were then followed up by their primary care physician and referred to the hospital when needed. Patients with <4 weeks of follow-up were considered lost to the follow-up.

A study protocol was written before data collection started, and prognostic variables were selected a priori based on perceived clinical importance: gender, age at injury, preoperative ulnar neuropathy, wound in relation to the fracture, fracture type (Mayo type 2A/2B), time from injury to fracture surgery, fixation method (TBW or PF), transcortical or intramedullary K-wire positioning (Fig. 1), and quality of postoperative reduction. Presence of wounds in relation to the olecranon fracture was classified as no wound (closed fracture), abrasion (no dermal perforation), or open fracture.

The radiographic examinations were reviewed by orthopedic surgeons and classified as two-part fracture (Mayo 2A) or multifragmented fracture (Mayo 2B). In cases where the fracture classification was uncertain, the senior author (KSM) reviewed and made the final decision on classification. K-wire fixation in TBW was classified as penetrating the anterior cortex (transcortical) or placed intramedullary. Reduction quality was assessed on noncalibrated lateral postoperative radiographs, and reduction quality was categorized as anatomically or nonanatomically (step and/or diastasis > 2 mm).^8^ Hardware failure was defined as secondary displacement leading to reoperation.

The reasons for secondary surgery were classified into the following categories: (1) Hardware-related pain, (2) hardware failure, (3) infection, (4) nonunion, (5) elbow stiffness, (6) misplaced hardware, (7) ulnar neuropathy, (8) malreduction, (9) wound dehiscence, and (10) routine removal (without symptomatic hardware). Reasons other than hardware-related pain and routine removal were considered major complications leading to secondary surgery.

If patients had multiple secondary surgeries, the initial complication causing secondary surgery was chosen (eg, if a patient underwent revision for infection and later for hardware failure, infection was chosen as primary reason for secondary surgery).

### Statistical analysis

The aim of this study was to build a predictive model for secondary surgery after olecranon fracture. Multivariable logistic regression models were constructed to assess the independent effect of numerous covariates determined a priori. Model complexity (number of predictors and number of spline knots) was determined using the rule of thumb that there should be no more than 1-model degree of freedom for every 15 events in the dependent variable.^9^ Multicollinearity was assessed using generalized variance inflation factors and bootstrap resampling was performed to assess model calibration. In addition, to understand the contributing factors associated with fixation method, a multiple logistic regression model was constructed to predict fixation method with age, sex, Mayo classification, and presence of a wound. All graphs and analyses were completed with the statistical package R, version 3.6.2 (R Development Core Team, Vienna, Austria with additional package rms; access date April 10, 2020).

## Results

**Figure 2:**
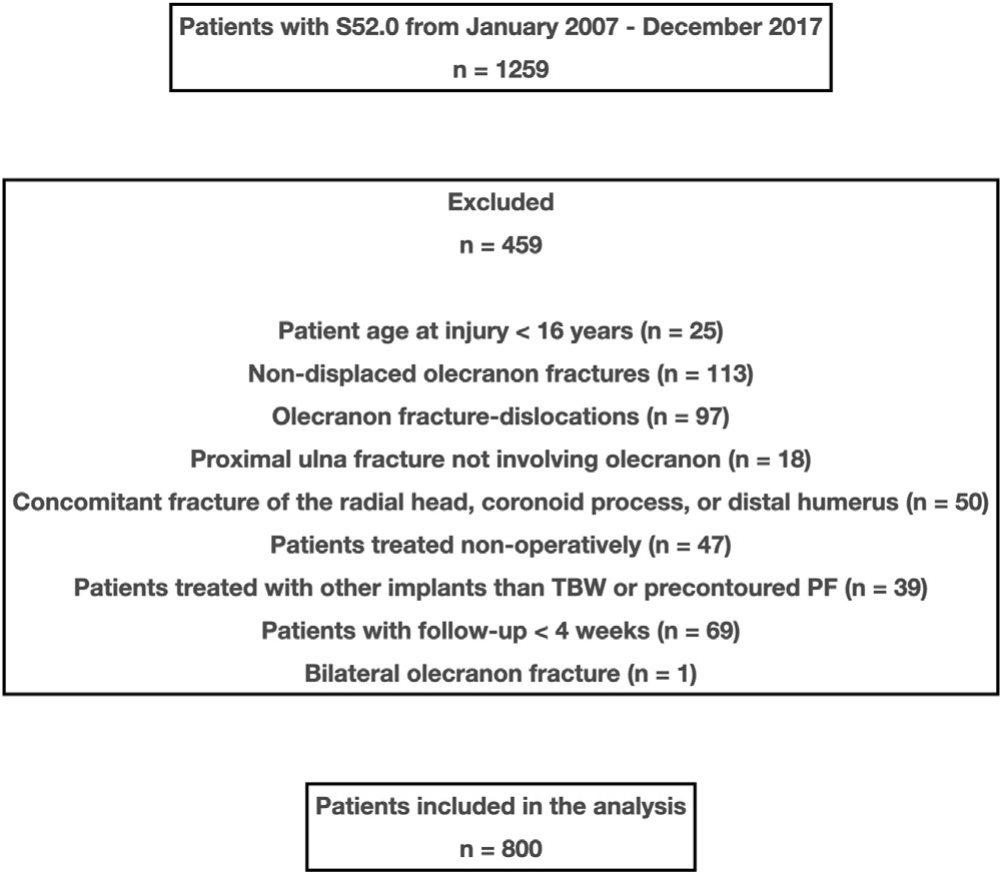
Summary of patients identified with isolated, displaced olecranon fractures treated with tension band wiring (TBW) and plate fixation (PF). Patients treated with other plate systems than precontoured plates such as reconstruction plates, one-third tubular plates, dual mini plates, and generic plates were excluded.

A total of 1259 patients with olecranon fractures were treated at seven Norwegian hospitals from January 2007 to December 2017. After application of inclusion and exclusion criteria, 800 olecranon fractures were included in the analysis (Fig. 2). There were 520 women (65%) and 280 men (35%), and the median age at injury was 62 years (range, 16-100) (Table I). There was an equal distribution of two-part (Mayo 2A) fractures and multifragmented (Mayo 2B) fractures. Of the 800 patients, 636 fractures were fixed with TBW and 164 fractures were fixed with PF (Table II). Following multivariable logistic regression analysis, adjusted variables influencing preference for PF were fracture classification (Mayo 2B) and lower age (Table III).

**Table I:** Study characteristics of 800 patients with isolated, displaced olecranon fractures treated with open reduction and internal fixation with tension band wiring (TBW) or plate fixation (PF).

Parameter	
Age at injury, median (range), yr	62 (16-100)
Median days from injury to operation (range)	2 (0-389)
Median follow-up time, weeks (range)	16 (4-528)
Gender	
Female (%)	520 (65)
Male (%)	280 (35)
Mayo type	
2A (%)	401 (50)
2B (%)	399 (50)
Abrasion or wound	
No n, (%)	644 (80)
Abrasion n, (%)	110 (14)
Open fracture n, (%)	46 (6)
Preoperative ulnar neuropathy	
No (%)	787 (98)
Yes (%)	13 (2)
Treatment	
TBW-AC (%)	585 (73)
TBW-IM (%)	51 (6)
PF (%)	164 (21)
Quality of reduction	
Anatomic (%)	596 (74)
Diastasis (%)	102 (13)
Step (%)	56 (7)
Step and diastasis (%)	46 (6)

*AC*, transcortical fixation of the K-wires through the anterior cortex of the ulna; *IM*, Intramedullary fixation of the K-wires.

**Table II:** Comparison of patient and injury characteristics of patients treated with tension band wiring (TBW) or plate fixation (PF).

Parameters	Total (N = 800)	TBW (N = 636)	PF (N = 164)	*P* value
Age at injury, median (range), yr	62 (16-100)	63 (16-100)	59 (16-98)	.02
Gender				.07
Female (%)	520 (65)	423 (81)	97 (19)	
Male (%)	280 (35)	213 (76)	67 (24)	
Mayo type				<.01
2A (%)	401 (50)	367 (92)	34 (8)	
2B (%)	399 (50)	269 (67)	130 (33)	
Abrasion or wound				.01
No (%)	644 (81)	525 (82)	119 (18)	
Abrasion (%)	110 (14)	80 (73)	30 (27)	
Open (%)	46 (6)	31 (67)	15 (33)	
Preoperative ulnar neuropathy				.82
No (%)	787 (98)	626 (80)	161 (20)	
Yes (%)	13 (2)	10 (77)	3 (23)	

NOTE. As per the Mayo classification, two-part fractures were classified Mayo 2A and multifragmented fractures were classified as Mayo 2B.

**Table III:** The odds ratio (OR) with 95% confidence interval (CI) and *P* value for factors influencing preference for plate fixation.

Parameters	OR	95% CI	*P* value
Age by 10 yr increments	0.90	0.82-0.99	.03
Male	1.04	0.69-1.56	.85
Mayo 2B	5.30	3.54-8.12	<.01
Abrasion	1.35	0.82-2.21	.23
Open fracture	1.78	0.87-3.54	.11

**Figure 3:**
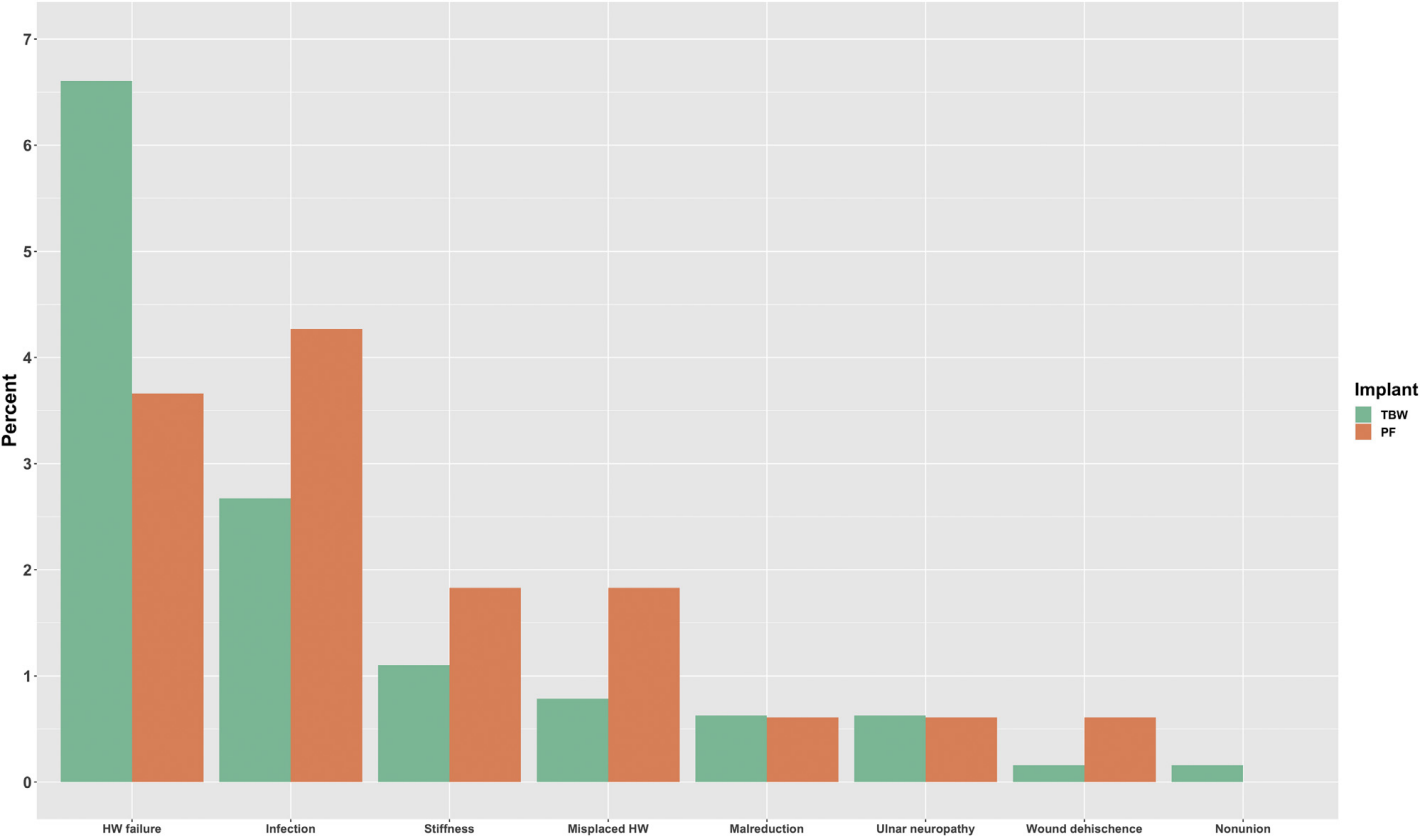
Summary of patients with major (hardware failure, infection, elbow stiffness, misplaced hardware, malreduction, ulnar neuropathy, wound dehiscence, and nonunion). *HW*, hardware; *TBW*, tension band wiring; *PF*, plate fixation.

Secondary surgery was performed in 329 of 800 patients (41%). Symptomatic hardware removal was the indication in 2 of 3 cases (Table IV). Five patients treated with TBW (<1%) and 1 patient treated with PF (<1%) underwent hardware removal in the absence of symptomatic hardware or any other complication. The aggregated rate of routine and symptomatic hardware removal was 31% in the TBW group and 18% in the PF group (*P* < .01). However, complications leading to secondary surgery other than symptomatic hardware and routine removal was 13% in both groups (*P* = .96), respectively (Fig. 3). Although hardware failure was observed more frequently after TBW, serious complications such as infection, stiffness, misplaced hardware, and wound dehiscence were observed more frequently after PF.

**Table IV:** Summary of primary indications for secondary surgery in 329 patients.

Parameters	Total (N = 800)	TBW-AC (N = 585)	TBW-IM (N = 51)	PF (N = 164)
Symptomatic hardware, n (%)	219 (27)	167 (29)	23 (45)	29 (18)
Hardware failure, n (%)	48 (6)	35 (6)	7 (14)	6 (4)
Infection, n (%)	24 (3)	17 (3)	-	7 (4)
Elbow stiffness, n (%)	10 (1)	7 (1)	-	3 (2)
Misplaced hardware, n (%)	8 (1)	5 (1)	-	3 (2)
Routine removal, n (%)	6 (1)	5 (1)	-	1 (1)
Malreduction, n (%)	6 (1)	4 (1)	1 (2)	1 (1)
Ulnar neuropathy, n (%)	5 (1)	4 (1)	-	1 (1)
Wound dehischence, n (%)	2 (0)	1 (0)	-	1 (1)
Nonunion, n (%)	1 (0)	1 (0)	-	-

*PF*, plate fixation; *TBW-AC*, tension band wiring with K-wires through the anterior cortex; *TBW-IM*, tension band wiring with intramedullary K-wires.

Patient age at injury (*P* < .01) and treatment method (*P* < .01) were identified as statistically significant independent risk factors for secondary surgery. Specifically, higher age at injury was associated with lower risk, whereas treatment with TBW increased the risk of subsequent surgery (Table V). The rate of secondary surgery was 44% and 32% after TBW and PF, respectively (*P* < .01). Secondary surgery after TBW was performed in 246 of 585 patients (42%) and 30 of 51 patients (59%) after transcortical and intramedullary K-wire positioning, respectively. In patients treated with TBW, intramedullary K-wire positioning was associated with higher risk of secondary surgery than transcortical K-wire positioning (odds ratio, 2.1; 95% confidence interval, 1.13-3.99; *P* = .02).

**Table V:** Adjusted risk factors associated with secondary surgery.

Parameters	OR	95% CI	*P* value
Age by 10 yr increments	0.74	0.68-0.80	<.01
Male sex	0.94	0.66-1.32	.71
Days to surgery	1.01	0.99-1.03	.18
Mayo type 2B	1.13	0.82-1.56	.45
Preoperative ulnar neuropathy	1.43	0.45-4.73	.54
Quality of reduction			
Diastasis	0.95	0.59-1.52	.83
Step	0.81	0.44-1.47	.49
Diastasis and step	1.41	0.73-2.70	.30
Tension band wiring			
K-wires through anterior cortex	2.06	1.36-3.14	<.01
K-wires intramedullary	4.32	2.16-8.86	<.01
Wound			
Abrasion	1.51	0.97-2.35	.07
Open fracture	1.2	0.62-2.29	.59

Odds ratio (OR) with 95% confidence interval (CI).

Days from injury to surgery, Mayo type, presence of preoperative ulnar symptoms, quality of reduction measured on postoperative radiographs, patient sex, and presence of abrasion or wound did not significantly influence risk of secondary surgery (all *P* > .05).

## Discussion

The principal findings of this study were that the rate of secondary surgery was 41% after fixation of isolated olecranon fractures with either TBW or precontoured PF. Based on 800 cases, the multivariable logistic regression model identified lower age at injury and treatment method as significant independent risk factors for secondary surgery. In regard to K-wire positioning in TBW, intramedullary K-wire positioning was associated with higher risk of secondary surgery than transcortical K-wire positioning. Interestingly, fragmentation of the fracture (Mayo type 2A/2B) did not influence risk of secondary surgery. The driver of the difference observed between the groups in secondary surgery was symptomatic hardware. The rate of reoperations due to major complications was similar between the groups.

Although olecranon fractures are considered a common elbow injury, only two randomized controlled trials have compared outcomes after TBW and PF – both without demonstrating significant differences in functional scores.^4^^,^^8^ Given that similar results after surgical treatment of isolated olecranon fractures can be expected, risk of secondary surgery is important when choosing treatment strategy. In the present study, symptomatic hardware removal was the dominating reason for secondary surgery, and this finding has been corroborated in several studies.^3^^,^^4^^,^^8^ Duckworth et al reported significantly higher rates of hardware removal after TBW than after PF (50% vs. 22%, *P* = .021). Hume and Wiss^8^ also found higher rates of symptomatic hardware after TBW (42%). These findings support the results of the present study as TBW significantly increased risk of secondary surgery.

Compared with PF, transcortical K-wire fixation doubled the odds ratio of secondary surgery, and intramedullary K-wire fixation was associated with more than four times the greater odds ratio. In a cadaveric biomechanical study, Mullett et al^15^ demonstrated that the average pullout strength of intramedullary K-wires and transcortical K-wires was 56.3 N (range, 27.7-95.6 N) and 122.7 N (range, 56.7-201.2 N), respectively. Saeed et al^17^ reported that the mean pullout was 2.4 mm and 5.5 mm in the transcortical and intramedullary K-wire groups, respectively (*P* < .01). As several studies have reported higher stability and lower rate of hardware migration with transcortical fixation,^15^^,^^20^^,^^21^ there seems to be sufficient evidence to avoid intramedullary K-wire positioning.

In the present study, increasing age was also found to reduce the risk of secondary surgery. As functional requirements decrease with higher age, our findings suggest that hardware prominence does not seem to require implant removal with increasing age. In a similar study of 392 patients, Claessen et al^2^ reported that the rate of secondary surgery was 25% of which the majority (93%) involved hardware removal. After multivariable regression analysis, secondary surgery was more frequently requested by women and younger patients. No association was found for diagnosed obesity, smoking, head injury, open or other fracture, Mayo type (2A/2B), implant type (TBW/PF), or TBW technique. The influence of patient age was corroborated in the present study, but we did not find any differences in gender. In contrast to Claessen et al, we found that implant type (TBW vs. PF) influenced rate of secondary surgery. This difference could be explained by the considerable sample size of the present study, even though Claessen et al reported 87% power in their study.

The quality of reduction on postoperative radiographs did not influence the rate of secondary surgery. This finding must be interpreted with caution as significant malreduction is easily overlooked. In a radiographic and cadaveric study, 27% of orthopedic trauma surgeons failed to identify a 5-mm central articular step on perfect lateral radiographs.^10^

There are several limitations to this study which have to be considered carefully when interpreting the results. The data were retrospective and collected from medical records and radiographs. Using the rule of thumb that there should be no more than 1 degree of freedom for every dependent variable, we chose nine variables that were considered clinically important. However, only about 1 in 5 patients were operated with PF. There may be a bias in patient selection or surgeon's preference that persists even after the regression analysis. Another limitation was that we used the ICD-10 code for identification of eligible patients. Eligible patients could have been missed, but this would not represent a systematic error that could skew the results. Seven hospitals participated in the study; 800 patients and 329 secondary surgeries were identified, thus providing sufficient power to detect small differences. However, our results must be interpreted observing that we did not measure the total number of complications, only the ones leading to a reoperation. For instance, it may be the case that elderly patients with symptomatic hardware were more reluctant to undergo a second procedure. It may equally be a lower threshold for patient or surgeon to remove the TBW than the PF owing to perceived risk of complications or the ease of the procedure. Another important limitation was the short follow-up in some patients owing to the fact that the majority of patients at the study center routinely are followed up for only one visit 4-6 weeks after surgery. However, Norway has universal health care and easy access in addition to follow-up when requested. Moreover, the Norwegian healthcare authority allocates patients to a specific hospital and the risk of secondary surgery being performed at a different institution was considered to be minimal. Finally, the postoperative radiographs were only reviewed by a single surgeon. Although the risk of failing to identify malreduction on radiographs is high, this risk is most likely inherent to the limitations of plain radiographs.

## Conclusion

Surgeons preferred to use PF in younger patients and multifragmented fractures. Patients should be counseled that secondary surgery is common after surgical fixation of olecranon fractures. Symptomatic hardware removal was the most frequently reported reason for secondary surgery and more frequent after TBW. When using TBW, intramedullary K-wire positioning should be avoided. The rate of major complications leading to secondary surgery was similar in the TBW and PF groups. Overall, the risk of subsequent secondary surgery was higher in younger patients and patients treated with TBW.

## Disclaimers:

*Funding:* No funding was disclosed by the author(s).

*Conflicts of interest:* The authors, their immediate families, and any research foundations with which they are affiliated have not received any financial payments or other benefits from any commercial entity related to the subject of this article.
